# Genetic Background and GxE Interactions Modulate the Penetrance of a Naturally Occurring Wing Mutation in *Drosophila melanogaster*

**DOI:** 10.1534/g3.113.007831

**Published:** 2013-11-01

**Authors:** Joseph Lachance, Lawrence Jung, John R. True

**Affiliations:** * Department of Genetics, University of Pennsylvania, Philadelphia, Pennsylvania 19104-6145; † Department of Ecology and Evolution, Stony Brook University, Stony Brook, New York 11794-5245

**Keywords:** *Drosophila melanogaster*, epistasis, genotype-by-environment interactions, incomplete penetrance, *vesiculated*

## Abstract

Many genes involved in producing complex traits are incompletely penetrant. One such example is *vesiculated*, an X-linked gene in *Drosophila melanogaster* that results in wing defects. To examine the genetic architecture of a complex trait (wings containing vesicles), we placed a naturally occurring variant into multiple autosomal backgrounds and quantified penetrance and expressivity at a range of developmental temperatures. We found significant epistasis, genotype-by-environment interactions, and maternal effects. Sex and temperature effects were modulated by genetic background. The severity of wing phenotypes also varied across different genetic backgrounds, and expressivity was positively correlated with penetrance. We also found evidence of naturally segregating suppressors of *vesiculated*. These suppressors were present on both the second and third chromosomes, and complex interactions were observed. Taken together, these findings indicate that multiple genetic and environmental factors modulate the phenotypic effects of a naturally occurring *vesiculated* allele.

The mapping of genotype to phenotype is central to developmental genetics and has important evolutionary consequences (Lunzer *et al.* 2005; Benfey and Mitchell-Olds 2008; Rockman 2008; Lehner 2013). Both recessivity and incomplete penetrance mask the effects of alleles, and this masking can potentially influence allele frequencies and the probability of fixation. Observed phenotypes depend on many factors, including environmental effects, genotype-by-environment (GxE) interactions, and epistatic interactions (Lewontin 2000; Chandler *et al.* 2013). For example, cancer susceptibility depends upon GxE interactions (Shields and Harris 2000), and epistatic interactions are known to occur between quantitative trait loci for wing shape in *Drosophila melanogaster* (Mezey *et al.* 2005). One example of epistasis involves the appearance of suppressors, whereby the effects of an allele at one locus are masked by genetic variation at a second locus. In addition, the effects of genes often are modulated by genetic background, as seen with *Egfr* and *scalloped* in *D. melanogaster* (Polaczyk *et al.* 1998; Dworkin *et al.* 2009; Chari and Dworkin 2013). Genotype-phenotype maps also are influenced by the positions of genes in developmental pathways (Stern 2010).

*Penetrance* refers to the proportion of individuals with a given genetic variant that show the expected phenotype, and *incomplete penetrance* refers to situations in which <100% of individuals manifest the expected phenotype. Similarly, *expressivity* refers to the severity of phenotypes that are associated with a mutant allele, and alleles that can yield a range of phenotypes are said to have *variable expressivity*. Note that variable expressivity refers to a general property of genotype to phenotype maps and it is not to be confused with RNA transcription (*i.e.*, gene expression). Incomplete penetrance can be viewed as a lack of developmental canalization. Many traits are associated with incompletely penetrant alleles, such as sterility due to the *Hybrid male rescue* gene in *Drosophila* (Aruna *et al.* 2009) and congenital scoliosis in humans (Sparrow *et al.* 2012). Penetrance can act as a nuisance parameter in human genetics, making it harder to detect associations in genome-wide association studies (Hirschhorn and Daly 2005). Multiple environmental and genetic causes underlie incomplete penetrance, such as thresholds in gene expression (Raj *et al.* 2010) and the presence of molecular chaperones (Carey *et al.* 2006). Penetrance can also reflect levels of genetic buffering (Gibson and Dworkin 2004). Although it is known that penetrance can be modified by environment and/or genetic background (Schmalhausen 1949), the relative importance of each of these factors and whether they interact is largely unknown. In addition, one can ask whether penetrance and expressivity are correlated. Do conditions that favor high penetrance also result in more severe phenotypes? In recent years there has been increased emphasis on the role of epigenetics (Bjornsson *et al.* 2004; Youngson and Whitelaw 2008; Javierre *et al.* 2010), and an open question is whether maternal or paternal effects influence the penetrance of alleles.

In a previous study, we placed a number of X chromosomes from natural *Drosophila melanogaster* populations into different autosomal backgrounds (Lachance and True 2010). One of these X chromosomes, *2214*, was associated with incompletely penetrant wing defects in non-native autosomal backgrounds. We observed clear bubbles of fluid in newly unfolded wings of flies containing *2214* X chromosomes. As these flies aged, these bubbles either flattened to become wrinkled wing-blades or they remained as vesicles.

In this study, we used complementation tests to determine that naturally segregating wing variants involved mutations in the *vesiculated* (*vs*) gene. *vesiculated* was discovered over 80 years ago (Evang 1925), and although the recombination and cytogenetic map positions of *vs.* are known, it has yet to be mapped to the DNA sequence level (Judd *et al.* 1972; Tweedie *et al.* 2009). Because of this, subsequent experiments required a classical genetics approach. The *vs^2214^* containing X chromosome was placed into multiple genetic backgrounds in a range of developmental temperatures, and by assessing the penetrance and expressivity of wing defects we were able to determine the extent to which *vesiculated* mutants are buffered from alleles at other loci and environmental effects. We also tested whether maternal and/or paternal effects modify penetrance and determined the chromosomal basis of naturally segregating suppressors of *vesiculated*. Together, these findings reveal details about the development and genetic architecture of a complex trait.

## Materials and Methods

### Stocks and construction of lines

The *2214* X chromosome was placed into multiple autosomal backgrounds, which allowed us to perform chromosomal level-analyses of epistatic and environmental effects. X chromosomes were derived from wild-caught and laboratory stocks of *D. melanogaster*. A wild-caught X chromosome from Tuscaloosa, Alabama (*2214*) resulted in abnormal wing phenotypes when in other genetic backgrounds but not when it was in its natural genetic background. This line was collected by R. Yukilevich in 2004. The *2214* X (*vs^2214^* containing) chromosome was used in the majority of experiments described in this paper. In addition, two X-linked candidate loci were used in complementation tests: *vesiculated* (*vs*) and *inflated* (*if*). *vs^1^ and if^3^* mutant lines were obtained from the Bloomington Stock Center (stocks 144 and 3960, respectively). A previous study suggested that the *vesiculated* locus is found in the 6B2-6B3 cytogenetic region (Judd *et al.* 1972; Tweedie *et al.* 2009). Because of this, we obtained an X-chromosome deficiency line for the cytogenetic region 6B2:6C4 (*Df(1)Exel6240*). The *Df(1)Exel6240* line was generated by Exelisis, Inc., and it also was obtained from the Bloomington Stock Center (stock 7714).

Four different autosomal backgrounds were used in this study: *6326*, *2214*, *Rum Cay*, and *Sudbury*. *6326* was derived from a mapping line from the Bloomington Stock Center (stock 6326). *2214* and *Rum Cay* were collected by R. Yukilevich in 2004, and *Sudbury* was collected by T. Merritt in 2005. The *Rum Cay* stock was collected in the Bahamas (latitude 23.38, longitude: −74.50), and the *Sudbury* stock was collected Ontario, Canada (latitude 46.49, longitude: −81.10). The *6326* and *Sudbury* lines were isogenized with balancers, whereas the *Rum Cay* line was produced by 10 generations of sib-mating. Because of this, flies sharing the same autosomal background effectively had identical or nearly identical genomes. The balancer stock *w^1118^*;*T(2;3)ap^Xa^*/*CyO:TM3* was used in the construction of lines and was obtained from the Bloomington Stock Center (stock 2475). Fourth-chromosome effects were not tested. Flies were cultured on standard corn meal/molasses/agar medium supplemented with antibiotics (either penicillin at 40 μg/mL or a mix of tetracycline and streptomycin at 63 μg/mL and 19 μg/mL, respectively).

### Complementation tests

**Figure 1:**
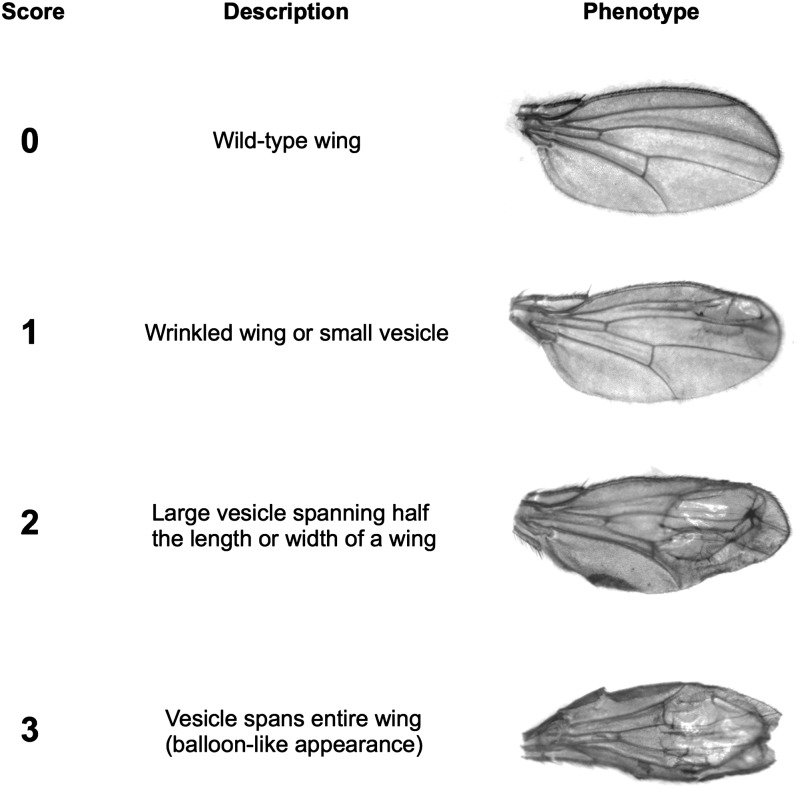
Exampes of wing vesicle phenotypes. Phenotypic scores are as follows: (0) wild-type wing, (1) wrinkled wing or the presence of a small vesicle less than half the width or length of a wing blade, (2) large vesicle spanning half the length or width of a wing blade, and (3) vesicle spans the entire wing, resulting in a balloon-like appearance.

To identify the genetic basis of naturally segregating variant (*vs^2214^*), we performed a number of complementation tests. The 2214 X chromosome contains a variant that is X-linked, completely recessive, incompletely penetrant, and results in wing defects that range from wrinkled wing blades to small bubbles or vesicles to balloon-like wings (Figure 1). These characteristics are shared with two candidate genes, *vesiculated* (*vs*) and *inflated* (*if*) (Lindsley and Zimm 1992). The *Df(1)Exel6240* X chromosome also was used in complementation tests to verify the approximate genomic region of the *vesiculated* gene. Heterozygous F1 females were generated for each complementation test, and the presence of wing defects was assessed. After denoting the *2214* X chromosome as *vs^2214^*, we performed four sets of complementation tests: *vs^2214^* with *vs^1^*, *vs^2214^* with *if ^3^*, *vs^2214^* with *Df(1)Exel6240*, and *vs^1^* with *Df(1)Exel6240*. To verify that a failure to complement was not due to epistatic autosomal effects, each complementation test was repeated in multiple autosomal backgrounds. For each test, 50 to 100 F1 females were phenotyped.

### Phenotypic assays

Wing phenotypes ranged from wild-type to wings with large vesicles encompassing an entire wing. Intermediate phenotypes involved a characteristic wing vesicle or blister (Figure 1). Because wings do not immediately unfold after eclosion and wing vesicles fray after 1−2 wk, flies were aged 3−5 d before being phenotyped. All flies tested in this section contained *vs^2214^* X chromosomes. Both sexes were scored for multiple combinations of genetic background (*6326*, *Sudbury*, and *Rum Cay*) and developmental temperature (17.5°, 20°, 21.5°, and 25°). Flies were mass mated and there were at least six vials per combination of treatments. For each wing, phenotypes were scored on a zero to three scale: (0) wild-type, (1) small vesicle or wrinkled, (2) large vesicle spanning half the length or width of a wing, and (3) vesicle encompassing entire wing giving a balloon-like appearance (Figure 1). Scoring of wing phenotypes was blind to the genotypes of flies. The number of flies scored for each combination of treatments (sex, background, and temperature) ranged from 107 to 367. Statistical tests for single factors and pairwise interactions involved a three-factor analysis of variance (ANOVA; fixed effects model). Note that genetic background treatments are classified as fixed effects because each set of autosomes is isogenic or near isogenic. Calculations were done using MATLAB (Mathworks 2005). Because flies with *Rum Cay* autosomes could not be maintained at 17.5°, data for this temperature were omitted from ANOVA calculations.

Because fluorescent lights can modify the penetrance and expressivity of wing defects (Pavelka *et al.* 1996), we examined whether this confounding factor had an effect on *vesiculated* mutants. For this test flies were grown at 25° in an incubator and placed 15 cm from a fluorescent light source. Light cycles were 12-hr light:dark. Half of the vials were wrapped in index cards, resulting in dark conditions. After waiting 4−9 d after eclosion, wing phenotypes were assayed. Fluorescent light experiments were replicated in two different lines: *vs^2214^*; *Sudbury*; *Sudbury* and *vs^2214^*; *Rum Cay*; *Rum Cay*.

Maternal and paternal effects were tested by crossing parents with different wing phenotypes. Four different types of crosses were performed: mothers and fathers with wing vesicles, mothers with wing vesicles and fathers with wild-type wings, mothers with wild-type wings and fathers with wing vesicles, and wild-type mothers and wild-type fathers. Parents and offspring of each cross were genetically identical, differing only whether they manifest an abnormal wing phenotype. For each cross, there were six replicate vials (with three females and three males per vial). Maternal and paternal effect experiments were replicated in two different lines: *vs^2214^*; *6326*; *6326* and *vs^2214^*; *Sudbury*; *Sudbury*. At least 370 F1 flies were phenotyped for each of these crosses. Developmental temperature for tests of maternal and paternal effects was 25°.

### Suppressor analyses

**Figure 2:**
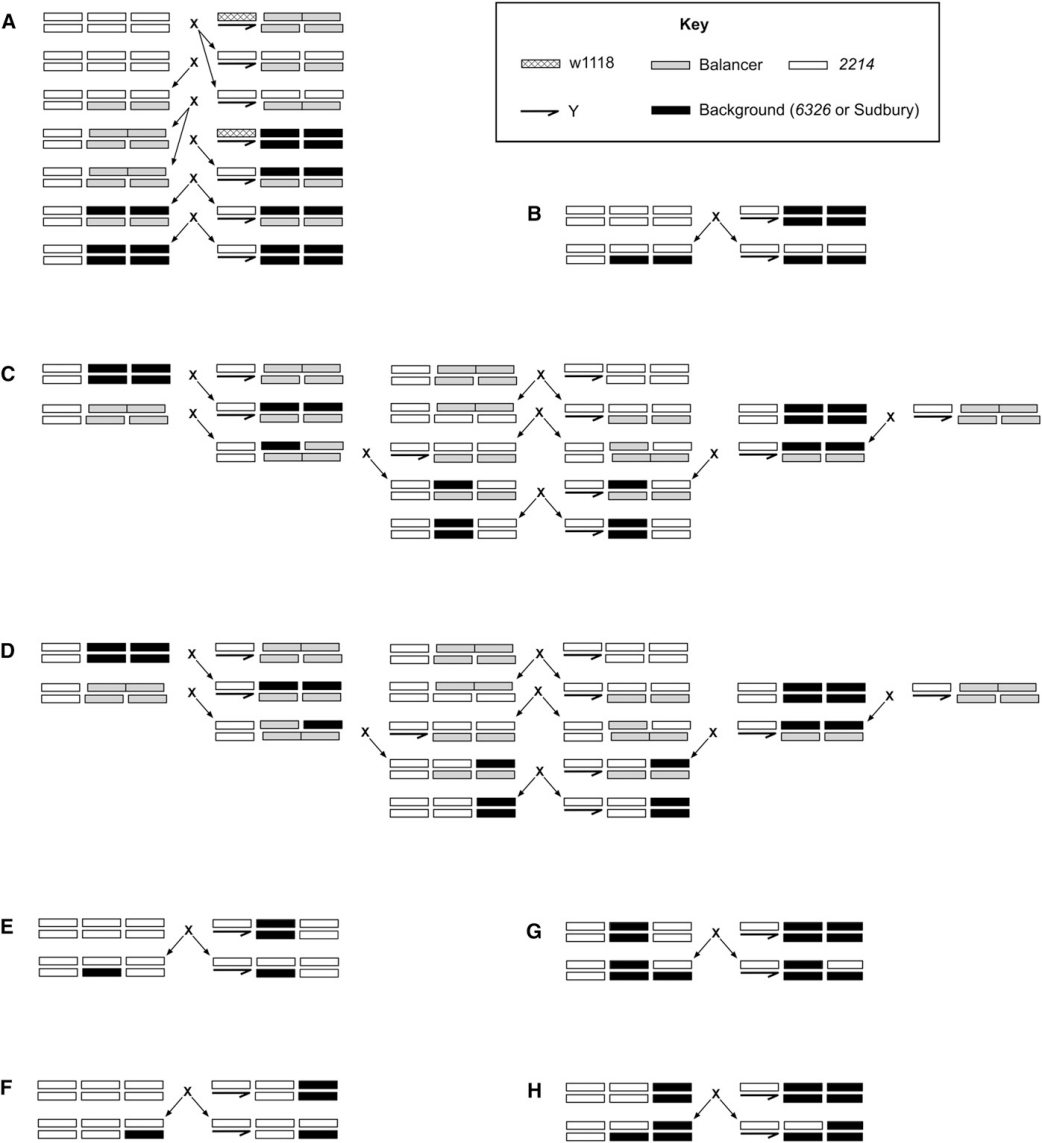
Crosses used to generate stocks for suppressor analyses. *2214* X chromosomes were placed into a number of different autosomal backgrounds. These sets of crosses were repeated for two different genetic backgrounds (*6326* and *Sudbury*). End results of crosses are as follows: (A) homozygous second and third chromosome; (B) heterozygous second and third chromosome; (C) homozygous second chromosome; (D) homozygous third chromosome; (E) heterozygous second chromosome; (F) heterozygous third chromosome; (G) homozygous second chromosome and heterozygous third chromosome; and (H) heterozygous second chromosome and homozygous third chromosome.

The *vs^2214^*-containing X chromosome does not exhibit abnormal wings in its natural genetic background. To test whether naturally occurring suppressors were on the second or third chromosome, we generated lines with mixed autosomal backgrounds (Figure 2). These genetic backgrounds contained a mix of *2214*, *6326*, and *Sudbury* autosomes. We also checked whether suppressors acted in a dominant or recessive fashion. All possible autosomal combinations were tested, and at least 55 flies of each sex were scored for each genotype. The 95% confidence intervals of proportions were calculated using the Agresti-Coull method (Agresti and Coull 1998).

## Results

### Complementation tests reveal that the observed wing defects are due to a mutation in the *vesiculated* gene

Complementation tests were performed using the *2214* X chromosome and the candidate genes *inflated* (*if*) and *vesiculated* (*vs*). Both of these recessive X-linked genes are known to result in incompletely penetrant wing defects (Lindsley and Zimm 1992). All F1 females heterozygous for the *2214* X chromosome and *if^3^* had wild-type wings, *i.e.*, these X chromosomes were able to complement each other. Conversely, approximately 25% of F1 females heterozygous for the *2214* X chromosome and *vs^1^* had wing vesicles, *i.e.*, there was a failure to complement. This failure to complement was observed in both *Sudbury* and *6326* autosomal backgrounds. Because the *2214* X chromosome failed to complement *vs^1^* but not *if ^3^*, it was designated *vs^2214^*.

Further complementation tests confirm that the *2214* X chromosome contains a mutation (or multiple mutations) in the approximate genomic position of the *vesiculated* gene. Inversion data from previous studies suggested that the *vesiculated* locus lies in the 6B2-6B3 cytogenetic region (Judd *et al.* 1972; Tweedie *et al.* 2009). Complementation tests of *vs^1^* and a deletion spanning 6B2:6C4 (*Df(1)Exel6240*) resulted in flies with wing vesicles (~25% of females). Similarly, *vs^2214^* showed a failure to complement the *Df(1)Exel6240* deletion construct in three different autosomal backgrounds. A similar inability to complement *Df(1)Exel6240* for *vs^1^* and *vs^2214^* reinforces the evidence that the 2214 X chromosome contains a mutation in the *vesiculated* gene. The X chromosome deletion in *Df(1)Exel6240* spans 125 kb and includes 10 genes.

### Penetrance varies by genetic background, sex, and temperature

**Figure 3:**
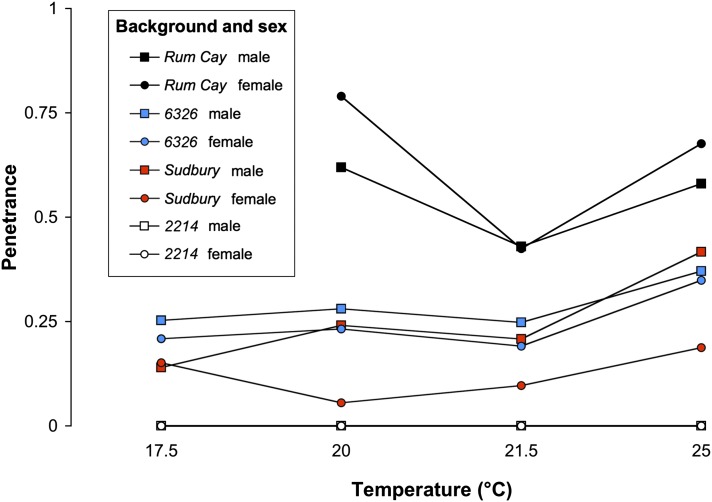
Penetrance reaction norms. The Y-axis indicates the proportion of flies containing wing vesicles. All flies tested contain X chromosomes with a *vs^2214^* genotype. Color indicates the autosomal background (*Rum Cay*: black, *6326*: blue, *Sudbury*: red, *2214*: white). Males are represented with squares and females with circles. Penetrance varies by autosomal background, sex, and developmental temperature. Note that temperature intervals are uneven and that *Rum Cay* flies were unable to be maintained at 17.5°.

As shown by the reaction norms in Figure 3, the proportion of flies with wing defects varied by each treatment. Recall that all of the flies tested in this section have *vs^2214^* X chromosomes but that they differ in their autosomal background, sex, and developmental temperature. Depending on the combination of treatments, penetrance of *vs^2214^* alleles ranged from 0 to 79%. A three-way ANOVA indicated significant effects of genetic background and temperature (Table 1). Penetrance was greatest for flies with a *Rum Cay* autosomal background (43–79%), and lowest for flies with a *2214* autosomal background (0%). A general trend was that penetrance was greater for flies grown at greater temperatures. For example, the penetrance in *6326* and *Sudbury* backgrounds was approximately 2% greater for each additional degree Celsius. In addition, there were clear interactions between temperature and genetic background (Table 1, Figure 3). Most notably, there was a nonlinear relationship between developmental temperature and the penetrance of flies with *Rum Cay* autosomes. Although there was no general sex effect, there were interactions between background and sex. In particular, female flies with *Rum Cay* autosomes had greater penetrance than male flies, whereas male flies with *6326* or *Sudbury* autosomes had greater penetrance than female flies. These sex-specific differences were on the order of 4% and 12%, respectively. Taken together, the reaction norms in Figure 3 indicate that penetrance is not an intrinsic characteristic of *vs^2214^* alleles. Instead, accurate estimates of penetrance require knowledge of genetic background, sex, and temperature.

**Table 1:** Three-factor ANOVA table of penetrance data

Source	Sum Sq.	d.f.	Mean Sq.	F	p Value
Background	10,681.2	3	3560.39	197.26	<0.001
Sex	63.8	1	63.77	3.53	0.109
Temperature	617.4	2	308.72	17.1	0.003
Background*sex	537.3	3	179.09	9.92	0.010
Background*temperature	723.9	6	120.66	6.68	0.018
Sex*temperature	8.7	2	4.36	0.24	0.793
Error	108.3	6	18.05		
Total	12,740.6	23			

The effects of autosomal background, sex, and developmental temperature were tested, as were pairwise interactions. Note that 17.5°, data were omitted from this test. Statistically Significant (p < 0.05) effects were observed for background, temperature, background*sex, and background*temperature. ANOVA: analysis of variance.

Further studies revealed that fluorescent lights moderately increased the penetrance of *vesiculated* mutants. Data from both sexes were pooled and the mean number of flies assayed per treatment was 158. *Sudbury* autosome-containing flies exposed to light had a mean penetrance of 8.1%, and flies kept in the dark had a mean penetrance of 4.5% (p = 0.270, two-tailed Fisher’s exact test). *Rum Cay* autosome-containing flies exposed to light had a mean penetrance of 96.2%, and flies kept in the dark had a penetrance of 60.0% (p = 0.0391, two-tailed Fisher’s exact test). The mechanism causing these patterns is unknown, and neither locomotor activity nor temperature differences between light and dark treatments can be ruled out.

### Expressivity data (severity of wing defects)

**Figure 4:**
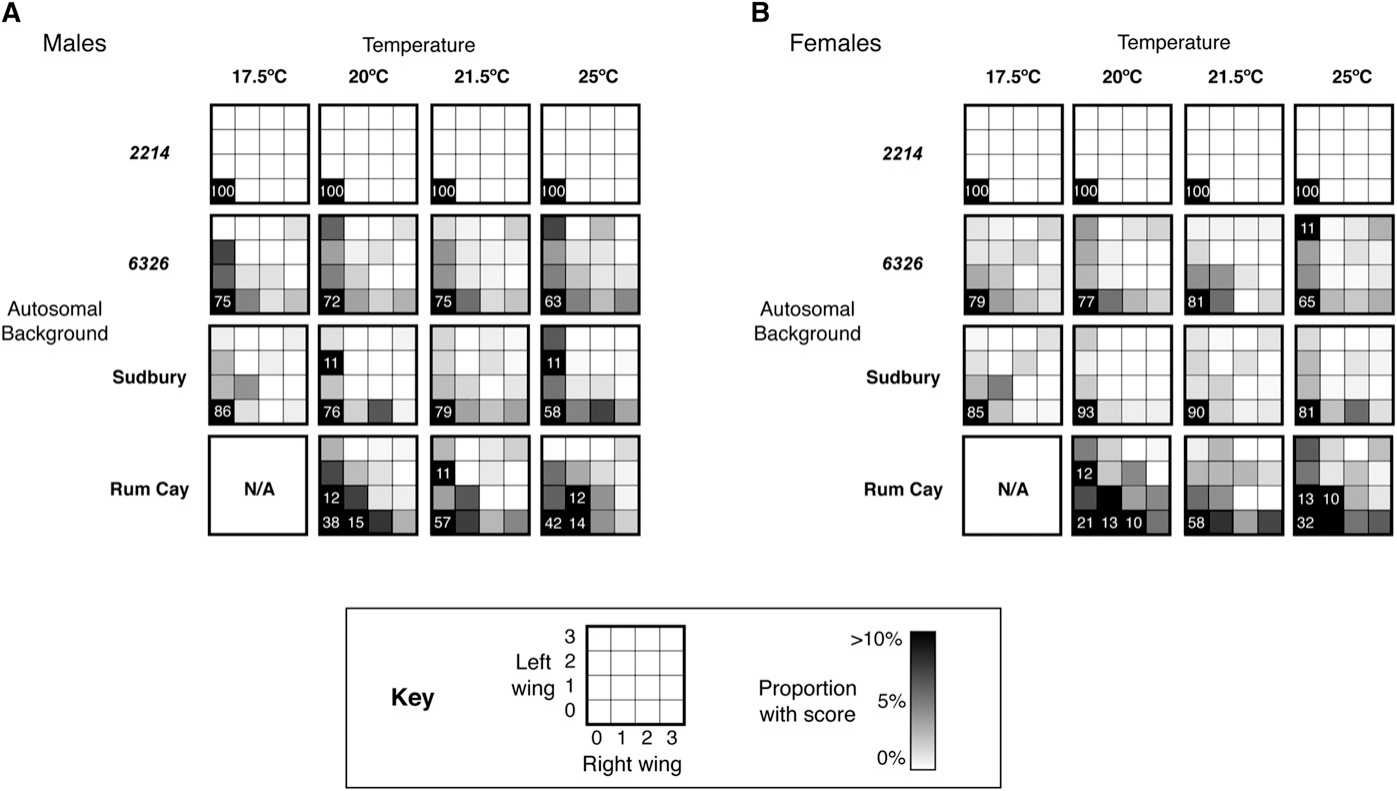
Expressivity of *vesiculated* in multiple genetic backgrounds. All flies tested have *vs^2214^* X chromosomes. Phenotypic scores range from 0 (wild-type) to 3 (balloon-like wings). For each treatment, shading in each cell of a 4 × 4 grid indicates the proportion of flies with a particular combination of left and right wing scores. Shading ranges from white (0%) to black (>10%). For scores >10%, the percentage is also listed. (A) Expressivity data for males. (B) Expressivity data for females. Median number of flies per combination of treatments (sex, temperature, and autosomal background) is 213.

Sex, genetic background, and temperature can influence not only presence or absence of wing defects but also the severity of wing defects. We examined flies with *vs^2214^* containing X chromosomes and quantified the severity of wing phenotypes for both sexes, four different autosomal backgrounds, and four different developmental temperatures. For each treatment, the proportion of flies with a particular combination of left and right wing scores is indicated with shading in Figure 4. Overall, autosomal background had a large effect on the severity of wing defects. Mean phenotypic scores varied by genetic background: 0.000 for *2214*, 0.343 for *6326*, 0.216 for *Sudbury*, and 0.647 for *Rum Cay*. Most flies with *Sudbury* or *6326* autosomes were wild type. In contrast, many flies with *Rum Cay* autosomes had wing vesicles, often in both wings. To a lesser extent, expressivity also varied by temperature (contrast rows and columns in Figure 4). Sex differences in the severity of wing phenotypes were minimal. Overall, there was no significant left-right asymmetry in the presence and magnitude of wing vesicles (p > 0.5, two sample Z-test). Also, the probability that one wing was defective was not independent of the probability that the other wing was defective (p < 0.00001, χ^2^ test of independence with 1 d.f.). We observed an overabundance of flies with both wings affected (4.49% compared with 1.57%, the product of left and right wing penetrance). This finding suggests that factors influencing the wing phenotypes of individual flies acted globally rather than locally.

After controlling differences in the presence or absence of wing defects (*i.e.*, omitting wings with phenotypic scores of 0), we found there was a positive correlation between the penetrance for a set of conditions (background, sex, and temperature) and the average phenotypic score of wings containing defects (*r* = 0.2884, p < 0.05, 2-tailed *t*-test). Conditions that increased the probability of observing wings with vesicles also increased the severity of wing defects when they occurred.

### Incomplete penetrance exhibits maternal effects

Flies were more likely to have wing vesicles if their mothers also had wing vesicles. Pooling *6326* and *Sudbury* backgrounds, the penetrance of *vs^2214^* flies that had mothers with wing vesicles was 33.1%, and the penetrance of flies with wild-type mothers was 26.8% (p = 0.003, two sample Z-test). However, when individual autosomal backgrounds were considered, significant maternal effects were only observed for the *6326* genetic background (p = 0.026 for *6326*, p = 0.067 for *Sudbury*, two-sample Z-tests). Although penetrance differed slightly for flies that had fathers with wing vesicles and flies that had fathers with wild-type wings, there were no significant paternal effects. Pooling *6326* and *Sudbury* backgrounds, the penetrance of *vs^2214^* flies that had fathers with wing vesicles was 30.3%, and the penetrance of flies with wild-type fathers was 28.8% (p > 0.25, two sample Z-test). Thus, the overall trend was that maternal effects modified penetrance, and this pattern varied by genetic background.

### Suppression of *vesiculated* involves complex epistasis

**Figure 5:**
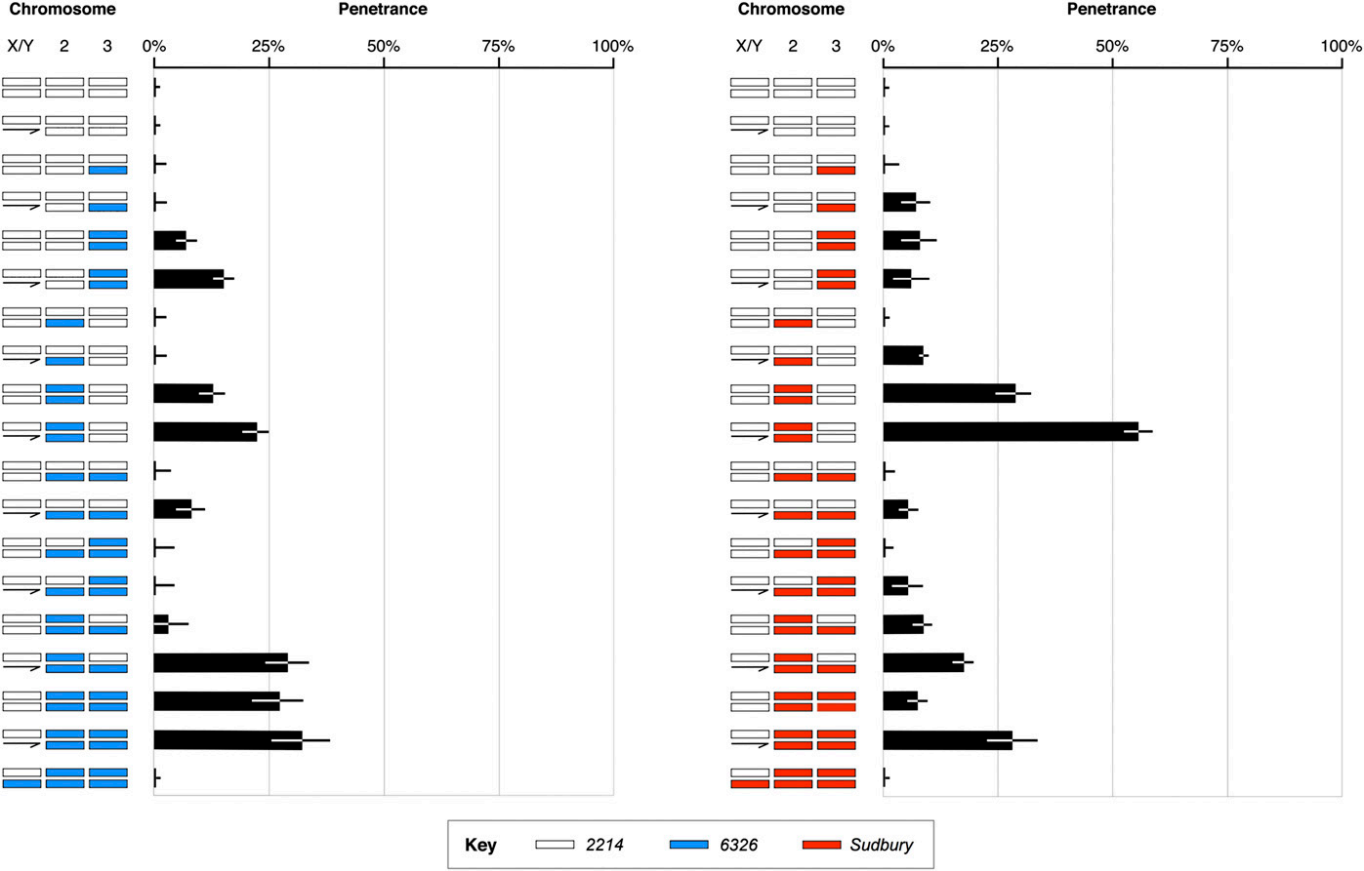
Suppression of *vs^2214^* by *2214* autosomes. Proportions of flies that had wing defects at 20° are depicted. Different chromosomes are represented by shading: *2214* in white, *6326* in blue, and *Sudbury* in red. Error bars indicate 95% confidence intervals.

The effects of *2214* X chromosomes can be masked by autosomes, and suppressors of *vs^2214^* were found on the second and third chromosomes. As indicated in Figure 5, flies with a *2214* autosomal background had wild-type wings. However, the presence of either *6326* or *Sudbury* autosomes resulted in flies with wing vesicles. Although some sex differences were observed, penetrance was largely determined by autosomal background. Flies homozygous for the *2214* third chromosome had greater penetrance than flies homozygous for the *2214* second chromosome (Figure 5), a finding that indicates that the *2214* second chromosome had a greater suppressive effect than the third chromosome. However, suppression of *vs^2214^* by *2214* autosomes was not additive, and complex patterns were observed (*i.e.*, penetrance of *vs^2214^* was not simply determined by the number of *2214* autosomes). In particular, flies with *2214* 2^nd^ chromosomes and *Sudbury* third chromosomes were more likely to have wing vesicles than flies with only *Sudbury* autosomes. Also, the effects of *2214* X chromosomes were different for different autosomal backgrounds (contrast the penetrance of flies with *6326* or *Sudbury* autosomes in Figure 5).

Suppression of *vs^2214^* by *2214* autosomes was partially dominant (Figure 5). In most cases, heterozygous genotypes containing *2214* autosomes had low penetrance. For example, the penetrance of males heterozygous for *2214* and *6326* autosomes was closer to that of males homozygous for *2214* autosomes than males homozygous for 6326 autosomes (8.0% *vs.* 0.0% and 28.0%). This dominance pattern was observed for both sexes and two genetic backgrounds (6326 and *Sudbury*).

## Discussion

Consistent with Schmalhausen’s view that penetrance and expressivity are the result of many environmental and genetic factors (Schmalhausen 1949), we find that penetrance is not an intrinsic property of the *vesiculated* gene. The presence of wings with vesicles was influenced by genetic background (GxG interactions), temperature (GxE interactions), and maternal effects. Importantly, temperature, sex, and maternal effects did not occur in isolation: they were modulated by genetic background. This underscores the importance of epistatic interactions, corroborating previous studies (Donehower *et al.* 1995; Atallah *et al.* 2004; Carlborg and Haley 2004; Phillips 2008; Dworkin *et al.* 2009). However, we note that our analysis was on a chromosomal level and that some of the observed effects may be due to interactions between autosomal loci and other X-linked loci than *vesiculated*.

The deletion construct used in this study suggests potential candidate loci for *vesiculated*. Genes within the 125-kb *Df(1)Exel6240* deletion include *CG34417*, *CG17717*, *Pat1*, *APC7*, *pigs*, *CG14443*, *CG3226*, *l(1)G0148Ctr1A*, *CG3224*, and *Ctr1A*. Similarly, the 6B2-6B3 cytological overlaps with the genes *dx* and *CG34417*. The gene *deltex* (*dx*) regulates Notch signaling (Yamada *et al.* 2011), and it is known to affect wing vein structure (Lindsley and Zimm 1992). A protein BLAST query reveals that the products of *CG34417* share sequence similarity with a Smoothelin cytoskeleton protein domain (Altschul *et al.* 1997). In humans, Smoothelin proteins colocalize with α-actin and are involved in contraction of smooth muscle cells (van Eys *et al.* 2007).

The physiology of wing development suggests possible mechanisms of *vesiculated* gene action. After eclosion, the wings of flies unfold due to an increase in hemolymph pressure (Johnson and Milner 1987). Wing hearts (lateral muscular pumps located in the thorax) then function as suction pumps that remove hemolymph from newly unfolding wings (Togel *et al.* 2008). During wing maturation, the wing cuticle delaminates, and components of the extracellular matrix are produced by wing epithelial cells (Kiger *et al.* 2007). These components include position-specific integrins and other molecules that allow the dorsal and ventral surfaces of the wing to bond (Brabant *et al.* 1996; Brown *et al.* 2000). *vesiculated* probably acts through one or both of these physiological processes (hemolymph pressure or adhesion of wing blade cells).

The patterns observed in this work allow some inferences about the genetic architecture of a complex trait to be made. For example, we observed an overabundance of flies with vesicles on both wings, suggesting developmental stochasticity acted on an organismal scale. It is unknown whether this global pattern of developmental stochasticity is a general characteristic of complex traits, or merely something that arises from wing vesicles (perhaps involving posteclosion hemolymph pressure as opposed to integrins and adhesion of individual wing blades). In addition, autosomal suppression of *vesiculated* involved multiple autosomes and complex epistasis. This finding indicates the presence of multiple modifier genes.

Our findings also are relevant to the topics of robustness and genetic buffering. Robustness refers to the ability of biological systems to function in the face of perturbations, and genotypes vary in their ability to buffer perturbations (de Visser *et al.* 2003; Kitano 2004; Wagner 2005; Masel and Siegal 2009). These perturbations are particularly important when phenotypic thresholds exist because continuous traits like gene expression can be converted into discrete phenotypes via development (Stern 2010). Gene expression above a threshold may result in a different phenotype than expression below a threshold. A study of intestinal cell fate in *Caenorhabditis elegans* revealed that incomplete penetrance can result from stochastic fluctuations in gene expression of unbuffered genotypes (Raj *et al.* 2010). Similarly, *vesiculated* mutants can be viewed as less buffered than wild-type alleles, as the effects of cryptic autosomal suppressors could only be seen in *vs^2214^* flies.

Importantly, the *vs^2214^* allele and *2214* autosomal suppressors segregate in natural populations. This adds to a growing body of literature that indicates that standing genetic variation can modify the effects of alleles (Wade *et al.* 1997; Polaczyk *et al.* 1998; Dworkin *et al.* 2003; Barrett and Schluter 2008). Although fitness effects in the wild are likely to be severe, the natural *vs^2214^* allele can segregate because of multiple reasons: it is recessive, it does not always manifest (effectively reducing any fitness effects), it is masked by naturally occurring suppressors, and phenotypic effects are modulated by temperature. Furthermore, population genetics theory indicates that recessive X-linked and autosomal alleles that combine to yield deleterious phenotypes can segregate at moderate frequencies (Lachance *et al.* 2011).

In contrast to a wealth of knowledge about genetic dominance and recessivity (Kacser and Burns 1981; Wilkie 1994; Kondrashov and Koonin 2004), relatively little is known about the basis of incomplete penetrance. One consideration is developmental noise. Stochastic effects are important when phenotypes are determined by a small number of molecules or cells (Raser and O’Shea 2005). If developmental noise causes gene expression to span both sides of a threshold, incomplete penetrance can result. Similarly, developmental stochasticity can be important when phenotypes are determined at a critical developmental time. Because wing unfolding only occurs once, chance events cannot be reversed. The position of a gene in a metabolic or developmental pathway may also affect whether mutations result in incomplete penetrance. There is evidence that genes at the center of hourglass-shaped pathways have large amounts of metabolic and/or developmental control (Kitano 2004; Stern 2010), and it is unknown whether these genes are more or less likely to be incompletely penetrant. It has also been hypothesized that Mendelian traits are more likely to involve completely penetrant variants, whereas complex traits are more likely to involve low penetrance variants (Antonarakis *et al.* 2010). A large number of genes modify wing shape in *D. melanogaster* (Zimmerman *et al.* 2000; Grieder *et al.* 2007), and we found that complex interactions underlie the penetrance of *vesiculated*. However, it is unknown whether a general pattern exists in which mutant forms of highly epistatic genes are more likely to be incompletely penetrant than genes with few interactions.

Here, we found that the penetrance of *vesiculated* alleles was a product of genetic background and environmental conditions rather than an intrinsic property of *vs^2214^* alleles. More broadly, general questions about penetrance can be asked. For example, do new mutations tend to have low or high penetrance? How evolvable is penetrance? Does a Haldanes’s sieve-like process yield a bias toward fixation of highly penetrant beneficial alleles? Because incomplete penetrance can influence allele frequencies and the probability of fixation, future studies of population genetics will benefit from theoretical models that allow alleles to be incompletely penetrant.
